# Reconceptualizing the balanced scorecard as a communication mechanism in healthcare

**DOI:** 10.3389/fpubh.2025.1623204

**Published:** 2025-07-21

**Authors:** Feng Guo, Ying Sophie Huang, Moeki Nemoto

**Affiliations:** ^1^School of Innovation and Entrepreneurship, Zhejiang Shuren University, Hangzhou, China; ^2^Department of Finance and Accounting, School of Management & Capital Market Research Center, Zhejiang University, Hangzhou, China; ^3^Faculty of Economics, The International University of Kagoshima, Kagoshima, Japan; ^4^School of Management, Zhejiang University, Hangzhou, China

**Keywords:** balanced scorecard, health communication, healthcare management, organizational communication, corporate communication, communication constitutes organization

## Abstract

The Balanced Scorecard (BSC) has been widely implemented in healthcare organizations primarily as a performance measurement system, yet its potential as a communication tool remains underexplored. This is particularly important given that communication failures have been implicated in over 70% of sentinel events in healthcare settings, making effective communication a critical determinant of patient safety and quality of care. This conceptual paper integrates micro-level communication constitutes organization (CCO) theory with macro-level corporate communication theory adapted for healthcare contexts to develop a multilayered model for analyzing BSC as a communication tool. Our framework consists of four interconnected layers: healthcare values foundation, BSC perspectives, healthcare communication processes (clinical strategic, interprofessional coordination, and healthcare stakeholder communication), and constitutive processes. We demonstrate the framework’s application through an electronic medical record implementation case and propose a Healthcare BSC Communication Matrix as a practical evaluation tool for healthcare leaders. This study reconceptualizes the BSC as a constitutive communication mechanism that can transform abstract healthcare values into measurable objectives, coordinate improvement efforts across disciplinary boundaries, and communicate progress to diverse stakeholders, offering both theoretical advancement for researchers and practical guidance for healthcare leaders seeking to enhance organizational communication through more effective BSC implementation.

## 1 Introduction

Effective communication lies at the heart of delivering high-quality healthcare and ensuring patient safety. Research consistently demonstrates that communication failures represent a leading cause of preventable adverse events in healthcare settings worldwide. The Joint Commission reports that communication failures were implicated in over 70% of sentinel events, making communication not only important but also critical to patient safety (1–3). Moreover, The Joint Commission’s 2023 Annual Review similarly identified communication failures, along with teamwork deficiencies and policy noncompliance, as the predominant root causes of sentinel events (4). This result underscores why communication must be viewed not merely as an optional skill but as a fundamental organizational competence essential to patient safety.

The importance of healthcare communication operates across multiple interconnected levels. At the patient-provider level, effective therapeutic communication enhances treatment adherence and patient outcomes (5). At the interprofessional level, standardized tools such as SBAR (situation, background, assessment, recommendation) bridge diverse communication styles, reducing errors by creating shared understanding across professional boundaries (1). Note that SBAR is a micro-level tool for standardizing clinical handoffs, and it is fundamentally different in both purpose and hierarchical level from the Balanced Scorecard (BSC), a management system at the organizational strategy level discussed later. At the organizational level, effective communication systems substantially reduce turnover. Structured onboarding communication strategies, in particular, have been shown to decrease new-hire turnover from 39.1 to 18.4% (6). In the case of the Bethlehem Garden Hospital, the turnover rate decreased from 23.6 to 3.4% through the operation of the BSC as a communication tool (7).

Recent research shows that communication in healthcare organizations has a direct impact on their financial performance. Creixans-Tenas et al. (8) empirically validated that strategic communication by hospitals has a positive effect on financial performance, establishing a statistically significant relationship between communication practices and economic outcomes. This indicates that effective communication contributes not only to improved quality of care but also to the financial sustainability of the organization.

Research based on relational coordination theory has established clear connections between communication quality and organizational outcomes. High-quality communication, characterized by frequency, timeliness, accuracy, and problem-solving orientation, positively impacts both staff well-being and patient satisfaction simultaneously (9, 10). During crises, the quality of leadership communication influences staff resilience, with a one-point improvement in communication measures associated with a 9% reduction in stress and a 19% reduction in burnout (11).

As Elrod and Fortenberry (12) emphasize in their work on integrated marketing communications in healthcare, institutions should consider communication as a strategic priority. Their research demonstrates that by implementing comprehensive and integrated marketing communication strategies, healthcare organizations can create synergies between various communication mechanisms, amplifying their impact and increasing the likelihood of achieving both communication goals and organizational outcomes. This integrated approach ensures that all marketing communications deployed by healthcare institutions present a cohesive picture to target audiences, enhancing both patient engagement and organizational performance.

Despite its recognized importance, multiple barriers impede effective healthcare communication. Hierarchical structures can create cultures of silence, where lower-ranking staff hesitate to voice their concerns (13). Nurses provide detailed, narrative reports, while physicians use concise, headline-style communication (1, 14). This mismatch can cause information gaps and misunderstandings.

Limited time, staff shortages, complex systems, and scattered information further complicate the challenging communication environment in healthcare. Healthcare professionals often waste precious minutes trying to find basic information, such as who to contact about a patient issue, how to reach that person, or what to do when they cannot be reached (2). This takes time away from actual patient care and adds to the stress and inefficiency of healthcare delivery.

As healthcare organizations grapple with staff retention, financial sustainability, and quality improvement, structured communication systems are no longer optional. Strategic management systems, such as the BSC, offer potential mechanisms to address these communication challenges while advancing organizational objectives.

While our previous research (15) established the BSC’s effectiveness as a corporate communication tool in a Japanese hospital setting, this paper advances the theoretical understanding by developing a healthcare-specific communication model. Traditional corporate communication frameworks, such as Van Riel and Fombrun’s (16) model with its management, marketing, and organizational communication clusters, do not adequately capture the unique communication dynamics in healthcare organizations. Healthcare contexts require specialized communication processes that address clinical priorities, interdisciplinary collaboration, and diverse stakeholder needs.

### 1.1 Research objectives and questions

This research pursues the following specific objectives:

1. To develop a theoretical framework that reconceptualizes the BSC from a performance measurement system to a constitutive communication mechanism in healthcare organizations.
2. To reveal, through this framework, how BSC transforms abstract healthcare values into measurable objectives, coordinates improvement efforts across disciplinary boundaries, and communicates progress to diverse stakeholders.
3. To provide practical guidance for healthcare leaders seeking to enhance organizational communication through more effective BSC implementation.

This study addresses the following key research questions:

- How does BSC function as a communication tool in healthcare settings?
- How do healthcare-specific communication processes influence BSC implementation and effectiveness?
- How can micro-level constitutive communication and macro-level strategic communication be integrated?

### 1.2 Methodological approach

This conceptual paper integrates two theoretical perspectives through the following systematic method:

1. Literature Analysis and Integration: Conducting a comprehensive review of communication constitutes organization (CCO) theory and corporate communication theory to identify the specificities of healthcare communication.
2. Theoretical Conceptualization: Developing key components of a healthcare-specific BSC communication framework based on empirical patterns identified in previous research.
3. Model Development: Creating a multilayered model illustrating bidirectional relationships between healthcare values, BSC dimensions, communication processes, and constitutive mechanisms.
4. Case Analysis: Analyzing published documents on Bethlehem Garden Hospital’s electronic medical record implementation to demonstrate the application of the proposed framework.

We integrate two complementary theoretical perspectives to create a more comprehensive analytical framework for understanding BSC as a communication tool in healthcare settings. At the micro-level, the CCO approach (17) examines how everyday communicative interactions constitute organizational reality through four flows: membership negotiation, organizational self-structuring, activity coordination, and institutional positioning. At the macro-level, we adapt corporate communication theory to propose three healthcare-specific communication dimensions: clinical strategic communication, interprofessional coordination communication, and healthcare stakeholder communication.

Communication in organizations can be understood through two fundamental yet complementary perspectives. At the micro-level, organizational communication theories (particularly the CCO approach) examine how everyday communicative interactions constitute organizational reality through messages, media, symbolic activities, and meaning-making processes (18). This perspective views communication not merely as something organizations do but as the very process through which organizations come into being and sustain themselves.

At the macro-level, corporate communication theory provides frameworks for understanding how organizations strategically manage communication with diverse stakeholders to achieve organizational objectives (19). This perspective focuses on the deliberate design and implementation of communication strategies, structures, and processes to enhance organizational performance and reputation. While micro perspectives reveal how communication constitutes organizations from the bottom up, macro perspectives illuminate how organizations intentionally manage communication from the top down.

As Thøger Christensen and Cornelissen (20) argue, these perspectives are not competing but complementary—the micro-level constructivist approach offers insights into how organizations emerge through communication, while the macro-level corporate approach provides frameworks for strategically managing these communicative processes. This complementarity is particularly relevant for analyzing BSC implementation, which involves both top-down strategic communication and bottom-up meaning-making processes simultaneously.

### 1.3 Theoretical and practical contributions of this research

By synthesizing micro-level CCO theory with macro-level corporate communication theory, this paper presents a multilayered model that illustrates the dynamic relationships between healthcare values, BSC dimensions, communication processes, and constitutive mechanisms. This paper contributes to both theoretical understanding and practical application in three key ways:

1. Bridging theoretical gaps: It reconceptualizes the BSC as a constitutive communication mechanism rather than merely a measurement tool, elevating scholarly understanding through a shift from performance measurement to organizational meaning-making.
2. Providing a healthcare-specific theoretical framework: It addresses the unique communication challenges faced by healthcare organizations, recognizing clinical priorities, interdisciplinary collaboration, and diverse stakeholder needs that influence BSC design and implementation.
3. Offering practical insights: It offers healthcare leaders actionable insights for leveraging BSC as a powerful communication mechanism that facilitates organizational transformation and improvement, bridging theory and practice to enhance both scholarly understanding and practical application.

### 1.4 Research scope and limitations

This study primarily focuses on long-term care healthcare institutions. It does not address in detail the specific challenges and opportunities in other healthcare settings, such as acute care hospitals, primary care, or specialty hospitals. Additionally, the proposed framework is based on the experience of a single Japanese hospital, Bethlehem Garden Hospital, and caution should be exercised when generalizing to environments with different healthcare systems, cultural backgrounds, or organizational structures.

This research draws primarily on experiences from East Asian healthcare environments in an international context. It does not explicitly address the specific challenges of BSC implementation in emerging market healthcare systems. Future research should test the framework’s applicability across various healthcare settings and geographical regions.

As this paper is a conceptual study aiming to develop a theoretical framework, it does not include empirical validation. Future research should collect and analyze multi-site data to test the proposed constitutive communication functions of the BSC in healthcare settings.

The paper proceeds as follows: Section 2 examines the four flows model in CCO theory and its application to BSC analysis. Section 3 develops a healthcare-adapted corporate communication framework. Section 4 presents our integrated framework for healthcare BSC communication. Section 5 provides an illustrative case demonstrating the practical application of the framework through Bethlehem Garden Hospital’s electronic medical record implementation. Section 6 presents a proposed evaluation framework. Finally, Section 7 discusses the implications and concludes.

## 2 The four flows model in CCO theory: a micro-level perspective for BSC analysis

The foundational concept underpinning CCO emerged in the 1980s, with the term “CCO” making its debut in a paper published by McPhee and Zaug in 2000 (21–23). Subsequently, Putnam and Nicotera provided concretization of the CCO role in their edited book, released in 2009 (24, 25). CCO is the idea that organizations are structured through communication, and it has had a major impact on organizational communication research (26). Within the CCO framework, three distinct schools of thought—the Montreal school, four flows, and social systems theory—offer diverse perspectives on communication (22, 23, 27). In relation to this study, the four flows approach is the preferred analytical framework. Since accounting, employee evaluation processes, and budgeting are explicitly addressed within the organizational self-structuring flow of this approach, it provides a valuable theoretical foundation for analyzing BSC as a communication tool in healthcare organizations.

The four flows approach posits that organizations are constituted through four distinct communicative flows: membership negotiation, organizational self-structuring, activity coordination, and institutional positioning (17). While rooted in structuration theory, this approach harbors its own unique tenets and interpretations (27). The four flows are described based on McPhee and Zaug (17):

1. Membership negotiation: This flow encompasses communication related to establishing, maintaining, and altering relationships with each organizational member. Communication to address identification, commitment, and personal positioning are examples of this flow (28). McPhee and Zaug (17) provide a specific example of recruitment and socialization.
2. Organizational self-structuring: Any communication process that directs the organization falls under this category. This includes policies, manuals, decision-making, budgeting, and accounting, to name a few. In other words, it involves interactions that generate rules and resources, directing the organization (23).
3. Activity coordination: This flow involves communication to coordinate work processes and resolve issues. Organizational strategy, negotiation, and hierarchical structure can serve as guidelines for activity coordination (28). The various actors in an organization are interdependent in accomplishing things and need communication to coordinate activities (26).
4. Institutional positioning: It pertains to communication that clarifies the organization’s position vis-à-vis clients, suppliers, competitors, and collaborators. Public and investor relations management are examples of this flow (22).

These four flows constitute essential prerequisites for organizational existence (22, 23, 26). This approach unveils the paradoxical facets inherent in an organization’s conditions of existence (e.g., orderly yet disorderly, cooperative yet competitive) (23).

The four flows approach has been studied in a wide range of research areas, including ISIL (29, 30), German startup companies (31), Tibetan niamles (32), and Danish engineering company (28) are examples. However, studies bridging the gap between CCO and accounting remain conspicuously scarce.

There are important theoretical and practical connections between the CCO approach and accounting practices. As Hines (33) argues, accounting practices do not merely describe organizational reality but actively construct it. This perspective is deeply relevant to BSC implementation.

Accounting practices, particularly strategic management accounting tools like the BSC, can be understood as central elements in the communicative constitution of organizations. As Huang and Nemoto (34) explain, management accounting communication heavily depends on codes and contexts, and is complicated by cognitive gaps between senders (management accountants) and receivers (managers). The BSC functions as a tool to bridge these gaps, facilitating a common understanding of organizational reality by translating strategic visions into measurable indicators.

The four flows model of CCO theory offers a new perspective for understanding accounting practices. As mentioned above, the organizational self-structuring flow explicitly mentions and positions accounting as a communication process that directs the organization (17). The language of accounting, especially frameworks like the BSC, becomes the means through which organizations define and redefine their boundaries, goals, and strategies. It is not merely a measurement tool but an organized system of communication that shapes and constitutes organizational identity and purpose.

This CCO perspective suggests that the BSC serves as an important communicative instrument within organizations. Its functioning can be better understood by examining how it operates across the four communicative flows identified in CCO theory. As Schoeneborn et al. (35) suggest, viewing tools like the BSC through this communicative lens helps us recognize organizations not as fixed entities but as processual entities that are continuously reconstituted through interconnected communicative practices.

Several meaningful connections emerge when examining the relationship between the BSC and the four flows model of CCO theory. The BSC can serve as a practical communication tool that facilitates all four communicative flows within an organization.

First, regarding membership negotiation, the BSC helps establish organizational identity by clearly communicating the organization’s mission, vision, and values. This communication creates a shared understanding among organizational members about what the organization stands for and what it aims to achieve. The learning and growth perspective of the BSC mainly addresses aspects of employee development, training, and engagement that directly influence how individuals identify with the organization.

Second, the BSC embodies organizational self-structuring through its role in formalizing communication processes. By establishing structured reporting relationships, performance indicators, and feedback mechanisms, the BSC generates rules and resources that direct the organization. The cause-and-effect relationships depicted in strategy maps provide a framework for decision-making processes, while the performance measures create accountability structures. The internal process perspective of the BSC focuses explicitly on the operational structures that define how work is conducted.

Third, about activity coordination, the BSC serves as a platform for aligning diverse organizational activities with strategic objectives. It facilitates cross-departmental coordination by creating a common language and shared understanding of priorities across functional areas. The BSC’s visual representation of strategy helps different departments understand how their activities contribute to overall organizational goals, potentially reducing silos and improving collaboration. However, while the BSC provides a framework for coordination, it may not address all the nuances of day-to-day communicative acts required for effective activity coordination.

Finally, concerning institutional positioning, the BSC helps organizations communicate their strategic position to external stakeholders. The financial and customer perspectives often address how the organization positions itself in relation to competitors, clients, and investors. When shared externally, the BSC can communicate the organization’s priorities, performance, and strategic direction to shareholders, partners, and other external stakeholders, thereby influencing how the organization is perceived in its institutional environment.

In addition, CCO provides a conceptual foundation for elucidating communication within non-profit organizations (36). For instance, American healthcare institutions achieved organizational self-structuring by adopting policies and procedures in response to the risk of Ebola hemorrhagic fever (37). Thus, the four flows approach proves efficacious even with healthcare institutions.

These connections demonstrate that the BSC can function as a comprehensive communication tool that addresses all four flows of the CCO theory. However, the effectiveness of the BSC in facilitating these communicative processes depends on how it is implemented and used within the specific organizational context. Organizations that actively use the BSC as a communication tool rather than merely a performance measurement system may experience enhanced organizational constitution through improved communicative practices.

## 3 Corporate communication framework: a macro-level perspective for BSC analysis

Building upon our previous research (15), which established the BSC’s effectiveness as a corporate communication tool in a Japanese hospital context, this section develops a healthcare-specific adaptation of corporate communication theory. While our earlier work adapted Van Riel and Fombrun’s (16) traditional framework to analyze BSC communication at Bethlehem Garden Hospital, this modification remained primarily within the bounds of generic corporate communication frameworks rather than developing a truly healthcare-specific approach. Such limitations meant that our framework could not adequately capture the unique communication dynamics inherent in healthcare settings, particularly the integration of clinical priorities with organizational strategy, the complexity of interprofessional collaboration, and the distinct requirements of patient-centered communication.

Corporate communication has the characteristic of organizing communication in an organization as a consistent entity (20). Early frameworks, such as those proposed by Grunig and Hunt (38), focused primarily on public relations models that described different approaches to managing communication with external stakeholders. Developments in the discipline have made corporate communication a framework that contributes to an organization’s competitive advantage through consistent communication with internal and external stakeholders (19).

Mohamad et al. (39) synthesize the definitions of corporate communication from nine previous studies, discerning three features: management instruments or tools, internal and external communication, and stakeholders or audiences. These features indicate that corporate communication is typically partitioned into internal and external communication. However, such a dichotomy is oversimplified and inadequate for analyzing organizational communication, particularly in healthcare settings. As Cornelissen (40) observes, organizations have diverse professionals with different communication needs and practices.

Traditional corporate communication frameworks, while useful for general organizational analysis, do not adequately reflect the unique challenges in healthcare contexts. Healthcare organizations have distinct communication requirements centered on clinical strategy and patient care outcomes. As we observed in our earlier study, the BSC’s role in facilitating the visualization of clinical strategy requires specific attention to communication processes that translate organizational strategy into patient care practices.

Additionally, the integrated nature of healthcare communication requires a framework that transcends traditional distinctions between marketing and organizational communication. Elrod and Fortenberry (12) emphasize how healthcare organizations benefit from integrated marketing communications where all promotional elements work cohesively rather than maintaining rigid divisions between different communication functions. Their research demonstrates that healthcare institutions must coordinate their verbal and visual expressions to create synergies across all communication channels, which enhances performance and increases the likelihood of achieving communication goals. This integrated approach is particularly relevant in nonprofit healthcare settings where building relationships with patients and communities takes precedence over traditional marketing objectives. They argue that such cohesion requires dedicated planning to align all communication elements with the organization’s desired imagery and appeals to target audiences.

Supporting this perspective, Creixans-Tenas et al. (8) found in their structural modeling study that hospital companies exhibit a clear relationship between social responsibility, communication practices, and economic and financial results. Their research validates that these three aspects are directly interrelated, suggesting that healthcare communication functions less as isolated marketing activities and more as integrated strategic components that simultaneously address stakeholder relationships, social responsibility, and organizational performance. This integrated approach supports moving beyond the conventional separation of marketing and organizational communication toward a more unified framework that better reflects the unique needs of healthcare’s stakeholder engagement.

Therefore, this paper proposes an adapted framework consisting of three healthcare-specific communication dimensions that refine and extend our previous application of corporate communication theory.

### 3.1 Definition of clinical strategic communication

Clinical strategic communication builds upon the broader concept of strategic communication, defined as “the purposeful use of communication by an organization to fulfill its mission” (41). Strategic communication is essential for managing and positioning an organization (42). In the healthcare context, clinical strategic communication adapts Van Riel and Fombrun’s (16) management communication by specifically focusing on the communication processes that connect an organization’s mission, vision, and patient care strategies directly to clinical practice.

This dimension is critical because, as previous research (1–3) has shown, communication failures represent a leading cause of adverse events in healthcare settings, contributing to over 70% of sentinel events. According to Joint Commission International Patient Safety Goal 2, effective communication between healthcare workers requires improvement to enhance patient safety (43). Research by McDermott et al. (44) further underscores the importance of formative cross-functional performance monitoring, showing that well-designed communication tools can positively influence both patient and employee outcomes through relational coordination.

While traditional management communication encompasses general organizational messaging, clinical strategic communication specifically focuses on translating organizational priorities into actionable clinical behaviors. This includes developing clear, logical narratives about quality and safety data, rather than presenting technical details. As Brown (45) shows, boards with high governance engagement receive concise, narrative-style reports that “tell the story” of clinical issues—linking the evidence base to current performance and planned interventions—thereby enabling nontechnical stakeholders to grasp complex quality problems and engage in meaningful challenges. Sangal et al. (11) demonstrated that effective leadership communication during the COVID-19 pandemic was associated with substantial reductions in both stress (9%) and burnout (19%) among frontline healthcare workers. This highlights how strategic clinical communication directly impacts provider well-being and, consequently, patient care quality.

This healthcare-specific dimension encompasses communication that directly supports the execution of clinical strategies, including quality improvement initiatives, the implementation of evidence-based practices, and the translation of organizational priorities into specific care practices. Examples include clinical strategy meetings, quality improvement forums, and multidisciplinary care conferences that align organizational objectives with patient-centered care delivery.

### 3.2 Definition of interprofessional coordination communication

Interprofessional coordination communication adapts Van Riel and Fombrun’s (16) management communication for healthcare’s unique multidisciplinary context. While their framework addresses general organizational communication, this dimension focuses specifically on communication processes that facilitate coordination among diverse healthcare professionals.

Traditional hierarchical structures in healthcare create a culture of inhibition and restraint in communication, as shown by Schmiedhofer et al. (13), rather than fostering a sense of open and safe communication. This underscores the need for specialized approaches to interprofessional communication.

Interprofessional coordination communication addresses the distinct communication challenges arising from professional differences. Research highlights how different healthcare disciplines develop varied communication approaches. Nurses typically employ narrative, contextual reporting styles, while physicians tend toward concise, action-oriented communication patterns (1, 14). These differences in professional communication can lead to information gaps and misunderstandings that affect patient care. To bridge these professional communication divides, standardized frameworks like SBAR have been implemented across healthcare settings, helping establish standard communication protocols that facilitate mutual understanding among diverse team members (1). Such structured approaches to interprofessional communication support the development of shared mental models that enhance team coordination and ultimately improve patient safety.

Research by Gittell et al. (9, 10) on relational coordination—defined by high-quality communication characterized by frequency, timeliness, accuracy, and problem-solving orientation—demonstrates that such coordination impacts both staff well-being and patient satisfaction simultaneously. This dimension includes communication mechanisms that enable different disciplines to develop shared understanding, coordinate patient care activities, and integrate diverse knowledge bases. Examples include interprofessional team meetings, structured handoff protocols, shared clinical documentation systems, and integrated care pathways.

### 3.3 Definition of healthcare stakeholder communication

Healthcare stakeholder communication combines and adapts Van Riel and Fombrun’s (16) marketing communication and organizational communication concepts for the healthcare context. While their framework separates product or service exchange communication from broader stakeholder relationships, in healthcare settings, these functions are integrated into communication processes that build trust relationships and facilitate information sharing with patients, families, community members, and healthcare-related organizations.

The importance of this dimension is underscored by research demonstrating that increasing transparency is crucial for healthcare institutions, as it promotes improvement in quality of care (46–48). Effective patient-centered communication is directly linked to improved health outcomes. As Kwame and Petrucka (5) explain, therapeutic communication enhances patients’ knowledge and understanding, builds trust, improves self-care skills, increases treatment adherence, provides comfort, and facilitates emotional management.

Healthcare stakeholder communication extends beyond traditional marketing approaches. Kulińska et al. (49) found that patients’ perceptions of safety and satisfaction were strongly correlated with information access, respectful treatment, and trust-building interactions. Their research demonstrates that communication serves as a mediating factor between technical medical care and patients’ experience of care quality. Satterstrom et al. (50) further emphasize the importance of creating transparent processes and communication channels that enable frontline staff to voice concerns and contribute to organizational improvement.

This dimension includes patient education initiatives, community health partnerships, regulatory compliance reporting, and transparent quality reporting. Examples include patient portals, community health forums, quality indicator publications, and partnership development activities that build trust with diverse stakeholders while advancing the healthcare organization’s mission.

This adaptation draws on Grodal et al.’s (51) concept of active categorization moves, reflecting our iterative process of refining theoretical categories based on empirical insights from our previous study. By critically examining the traditional communication divisions in the Van Riel and Fombrun (16) framework against our observations of BSC communication in healthcare settings, we develop a framework better suited to capture the role of the BSC as a communication tool in healthcare environments.

To complement this healthcare-adapted corporate communication framework, we also employ the four flows model from CCO theory (17). In the next section, we develop an integrated communication framework that combines these macro-level strategic communication management dimensions with micro-level constitutive processes. This integration creates a comprehensive analytical approach that better captures the complex communication dynamics inherent in healthcare organizations implementing BSC.

## 4 Integrated framework for healthcare BSC communication

The integration of corporate communication theory and CCO theory creates a comprehensive analytical framework for understanding how BSC functions as a communication tool in healthcare settings. This section presents a multilayered model that illustrates the dynamic relationships between healthcare values, BSC dimensions, communication processes, and constitutive mechanisms.

To clarify the distinct yet complementary roles of the two theoretical pillars supporting our framework, Table 1 compares their key characteristics and shows their specific contributions to each framework layer. This demonstrates how micro-level CCO theory and macro-level corporate communication theory work together to create a more comprehensive understanding than either theory could provide independently.

**Table 1:** Comparison of the two theoretical pillars: CCO and corporate communication (source: compiled by the authors).

Aspect	CCO theory	Corporate communication theory	Integration benefit
Analytical level	Micro-level constitutive processes	Macro-level strategic management	Bridges strategy and daily practice
Primary contribution to the framework	Constitutive processes layer (four flows)	Healthcare communication processes layer (three dimensions)	Integrates the constitutive process (how) with the strategic purpose (why) of communication
Key strength	Explains how communication creates reality	Explains strategic communication management	Combining emergence with intention
Application to BSC	Shows how BSC constitutes an organization	Shows how BSC manages stakeholder communication	BSC as both a constitutive and a strategic tool

### 4.1 Framework structure and relationships

**Figure 1:**
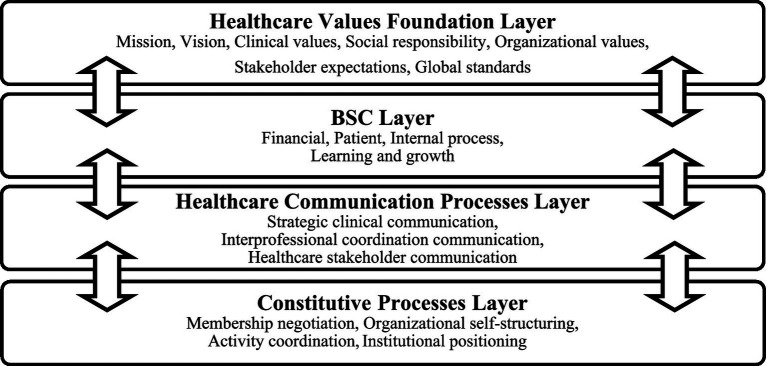
Healthcare BSC communication integration framework: A multilayered model (source: compiled by the authors). Each layer is interconnected through bidirectional arrows, indicating a continuous feedback loop. The framework integrates macro-level healthcare communication processes (third layer) with micro-level constitutive processes (bottom layer) to show how strategic initiatives are enacted and shaped through daily communication.

The integrated framework presented in Figure 1 consists of four interconnected layers that reflect the complex communication dynamics in healthcare organizations implementing BSC.

First, the healthcare values foundation layer represents the fundamental values that guide healthcare delivery. This layer encompasses six interconnected elements that collectively define the normative foundation for healthcare delivery:

1. Mission and Vision: Core organizational purpose and aspirational future state.
2. Organizational values: Fundamental beliefs about how care should be delivered (e.g., compassion, dignity, excellence).
3. Clinical values: Evidence-based care principles and quality standards.
4. Social responsibility values: Commitment to community health, equity, and accessibility.
5. Stakeholder expectations: Requirements from patients, regulators, community, and staff.
6. Global standards: Integration with frameworks like the Sustainable Development Goals (SDGs) for broader societal impact.

These elements serve as the foundation upon which all organizational communication is built and provide the normative context for BSC implementation.

Second, the BSC layer operationalizes these values through the four traditional BSC perspectives: financial, patient (adapted from customer), internal process, and learning and growth. This layer transforms abstract values into measurable objectives and indicators that can be communicated throughout the organization.

Third, the healthcare communication processes layer adapts traditional corporate communication theory to healthcare contexts through three dimensions:

- Clinical strategic communication: Connects organizational strategy to clinical practice
- Interprofessional coordination communication: Facilitates collaboration and knowledge integration among diverse healthcare professions
- Healthcare stakeholder communication: Builds trust and facilitates information sharing with diverse stakeholders

Fourth, the constitutive processes layer, derived from CCO theory, explains how BSC communication constitutes organizational reality through four flows:

- Membership negotiation: Strengthens identification with organizational values
- Organizational self-structuring: Creates coherent decision-making frameworks
- Activity coordination: Aligns diverse professional activities
- Institutional positioning: Enhances external relationships and reputation

### 4.2 Dynamic interactions between layers

The framework’s analytical power emerges from the complex bidirectional relationships between layers, represented by double-headed arrows in Figure 1. Rather than operating in isolation, these layers engage in continuous feedback loops that collectively enhance the BSC’s effectiveness as a communication tool in healthcare settings.

#### 4.2.1 Healthcare values foundation and BSC layer interaction

The healthcare values foundation layer provides the normative context from which BSC design emerges, while BSC implementation simultaneously reinforces and evolves these foundational values. For example, if a hospital integrates a core organizational value like “compassionate care” into specific patient perspective indicators, it transforms abstract values into measurable objectives that staff can understand and implement. Similarly, when sustainability goals, such as the SDGs, are incorporated into BSC indicators, they become operational priorities rather than aspirational statements.

This bidirectional relationship enables organizational learning about core values. As BSC indicators reveal performance gaps in areas such as patient-centered care or community service, the organization can refine its understanding of what these values mean in practice. For example, when a hospital discovers through BSC measurement that its free care programs reach fewer community members than intended, it might develop a more nuanced understanding of its social responsibility values and design more effective outreach initiatives.

#### 4.2.2 BSC layer and healthcare communication processes layer interaction

The BSC layer provides structured content for organizational communication, while the healthcare communication processes layer determines how this content is effectively disseminated, interpreted, and internalized. The BSC transforms abstract strategies into specific objectives and indicators, while the communication processes determine whether these objectives are understood consistently across diverse professional groups.

Clinical strategic communication ensures that financial and operational indicators are interpreted within the context of patient care priorities. For instance, when bed utilization indicators are communicated through clinical leadership meetings, they become not just operational targets but clinically meaningful indicators of care accessibility. Without this translation, clinicians might view such indicators as purely administrative concerns disconnected from patient care.

Interprofessional coordination communication addresses the distinct challenge of knowledge integration across professional boundaries. BSC can provide a common language that helps diverse specialists develop a shared understanding of organizational priorities. For example, when nursing, pharmacy, and physician teams track medication reconciliation indicators through a shared BSC dashboard, they develop collective accountability that transcends departmental boundaries.

Healthcare stakeholder communication extends BSC communication beyond organizational boundaries to patients, families, and community partners. When quality indicators are transparently shared with patients through accessible formats, the BSC becomes a tool for building trust and managing expectations. This transparency, in turn, creates external accountability that can drive internal performance improvement efforts.

#### 4.2.3 Healthcare communication processes and constitutive processes layer interaction

The healthcare communication processes layer operates through the mechanisms of the constitutive processes layer, while constitutive outcomes reshape communication practices over time. This relationship explains how formal communication structures influence the emergent realities of organizational life.

Clinical strategic communication primarily relies on membership negotiation flows to strengthen healthcare professionals’ identification with organizational values, but also interacts with other constitutive flows. When physicians participate in developing clinical quality indicators for the BSC, they experience greater alignment between their professional identity and organizational membership. This enhanced identification facilitates more authentic clinical strategic communication in subsequent interactions. Simultaneously, this participation process shapes decision-making frameworks through organizational self-structuring flows and facilitates practical implementation through activity coordination flows.

Interprofessional coordination communication primarily depends on activity coordination flows to align diverse professional activities, but also leverages organizational self-structuring flows. When multiple departments track shared BSC indicators for integrated care pathways, they can develop coordination patterns that transcend traditional hierarchical communication. These emergent coordination patterns reshape formal interprofessional communication structures, creating more efficient information flows. At the same time, this coordination influences cross-departmental decision-making processes through organizational self-structuring and strengthens collaborative interprofessional identity through membership negotiation.

Healthcare stakeholder communication primarily operates through institutional positioning flows to establish the organization’s relationships with external entities, but also connects with other constitutive processes. When a hospital shares its community health initiatives through BSC reporting, it positions itself as a community partner rather than merely a service provider. This positioning influences how the organization communicates with community stakeholders, creating expectations for ongoing transparency and collaboration. Simultaneously, this external communication is aligned with internal processes through organizational self-structuring and strengthens employees’ organizational identity and sense of purpose through membership negotiation.

Importantly, each of these communication dimensions does not exclusively relate to a single constitutive flow but rather interacts with all four flows to varying degrees. For instance, clinical strategic communication about patient safety initiatives might simultaneously impact membership negotiation (strengthening professional identity), organizational self-structuring (establishing safety protocols), activity coordination (aligning safety practices across departments), and institutional positioning (communicating safety commitments to patients and regulators). This complex interplay explains how BSC functions as a communication tool that can express and shape multiple organizational dimensions simultaneously.

### 4.3 Innovation as an emergent property of the framework

Innovation emerges through the dynamic interactions between framework layers. The BSC communication framework supports innovation through three key mechanisms:

1. Cross-boundary knowledge integration: Interprofessional coordination and communication facilitate the sharing of diverse professional knowledge, creating conditions for innovative solutions to emerge from the intersection of different disciplinary perspectives.
2. Continuous feedback loops: The bidirectional relationships between layers create systematic feedback mechanisms that identify opportunities for improvement and support iterative innovation processes.
3. Value-driven innovation: By grounding innovation efforts in healthcare values, the framework ensures that innovations align with organizational mission while addressing real patient care needs.

## 5 Illustrative case: electronic medical record implementation at Bethlehem garden hospital

The integrated nature of these dynamic interactions can be illustrated through Bethlehem Garden Hospital’s implementation of an electronic medical record (EMR), a real initiative executed within their BSC framework. This case illustrates how the multilayered communication model operates in practice. This case illustration is constructed entirely from previously published sources (15, 52–54) rather than new qualitative research conducted specifically for framework development. It represents a synthesis of documented experiences to demonstrate the practical application of the multilayered model. Of the descriptions based on each material, those presented in Japanese were translated by the authors.

Bethlehem Garden Hospital is guided by a principle that represents its core mission and vision, a key component of the healthcare values foundation layer: “We inherit the will of Father Joseph Flaujac, our founder, and faithfully provide warm medical care for the sick according to the spirit of Christ’s love.” Based on this foundation, the hospital identified improving business efficiency as a key strategic priority. This priority was formalized in their BSC (internal process perspective) as “business efficiency” with the specific outcome of implementing an electronic medical record system (15).

These objectives were communicated through multiple channels aligned with the healthcare communication dimensions identified in the framework. For clinical strategic communication, regular presentations by the Director shared the strategic context. For interprofessional coordination communication, the hospital established a project meeting structure that facilitated collaboration. For healthcare stakeholder communication, the hospital shared progress updates through its PR magazine, “Bethlehem Wind.”

As documented by Kikuchi (54), this project “began in earnest in FY 2018 and took 3 years to complete, with the system going live in December 2020.” The project team comprised approximately 20 staff members drawn from all hospital departments. As he noted:

The project team was responsible for almost all processes up to the introduction of the system, including selection of an electronic medical record manufacturer, consideration of necessary specifications, coordination of visits to medical institutions that had already introduced similar systems, determination of whether or not related departmental systems were required, contact for quotation negotiations, investigation and procurement of the necessary number of terminals, and lectures on operation methods.

Through these communication processes, the constitutive flows became evident. Membership negotiation occurred as staff from different departments collaborated on the project. As quoted in the PR magazine, a first-floor ward nurse experienced that “The text is now easier to read and more organized, which makes information easier to retrieve. Additionally, information sharing has become more seamless, facilitating better interprofessional collaboration” (52).

Organizational self-structuring emerged through the project meeting structure where, as Kikuchi (54) described: “The administrative side, consisting of the Director, the Nursing Director, and the Administrative Director, participated in the project meetings as observers and provided necessary support, mainly in terms of securing personnel and budget.”

Activity coordination materialized through the collaborative development of new workflows. As documented by Kikuchi (54):

The results of the monthly financial statements are disclosed to all employees at the monthly general meeting. Both full-time and part-time staff know whether there was a surplus or deficit this month in units of one thousand JPY. This accumulation of information is considered to have led the members of the project meeting to judge the balance between necessary specifications and installation costs independently and to make the best choice in selecting the electronic medical record system this time.

Institutional positioning developed as the hospital communicated these improvements to external stakeholders. The impacts extended beyond operational efficiency to enhance interprofessional communication. A second-floor ward nurse observed that “The most significant change after introducing the electronic medical record system is that information can now be shared across different professional groups. Through this shared information, we can observe how other professionals interact with patients. This allows us to learn about different aspects of each patient and thereby improve our interactions with them” (52). This staff voice is disseminated to external stakeholders through a PR magazine.

As the Administrative Director noted in Nikkei Healthcare (53), with the EMR implementation, “the handover process that used to take 10–15 min has been eliminated, allowing that time to be devoted to patient care.” This outcome demonstrates how the BSC’s communication framework translated strategic priorities into practical improvements in care delivery.

This case illustrates how a single BSC initiative at Bethlehem Garden Hospital operated across multiple framework layers simultaneously. The BSC provided not just a measurement tool but a communication mechanism that translated strategic priorities into operational practices, coordinated cross-professional activities, and facilitated bottom-up innovation. The initiative led to a more efficient and collaborative healthcare organization through these interconnected communication processes, as documented in the hospital’s experience.

## 6 Proposed evaluation framework

Building upon our integrated theoretical framework, we propose a practical evaluation tool for healthcare organizations seeking to enhance their use of BSC as a communication mechanism. This Healthcare BSC Communication Matrix (Table 2) translates the theoretical concepts from our multilayered model into an actionable framework that healthcare leaders can use to assess and improve their communication practices.

**Table 2:** Healthcare BSC communication matrix [source: compiled by the authors].

Strategic objective: [enter your strategic objective here]			
Communication Flows	Clinical strategic communication	Interprofessional coordination communication	Healthcare stakeholder communication
Membership Negotiation	Guideline: Develop strategic narratives that connect organizational priorities to professional identities Indicator: Professional identity alignment metrics (e.g., training participation, utilization rates) BSC: Learning & Growth	Guideline: Design collaborative processes that foster joint ownership across disciplines Indicator: Cross-disciplinary engagement metrics (e.g., workshop participation, feedback submission) BSC: Learning & Growth	Guideline: Articulate connections between stakeholder initiatives and staff’s core professional values Indicator: Value alignment metrics (e.g., staff adoption of patient-centered practices) BSC: Patient
Organizational Self-structuring	Guideline: Establish formal decision processes aligned with strategic clinical priorities Indicator: Process efficiency metrics (e.g., decision time, protocol adherence) BSC: Internal Process	Guideline: Create interdisciplinary structures that formalize coordination mechanisms Indicator: Structural effectiveness metrics (e.g., cross-unit information flow) BSC: Internal Process	Guideline: Implement systematic processes for integrating stakeholder feedback Indicator: Feedback utilization metrics (e.g., stakeholder input implementation rate) BSC: Patient
Activity Coordination	Guideline: Synchronize operational activities with strategic implementation timelines Indicator: Strategic alignment metrics (e.g., milestone achievement, resource utilization, implementation time for strategic innovations like EMR) BSC: Internal Process	Guideline: Develop standardized protocols for cross-functional information exchange Indicator: Coordination effectiveness metrics (e.g., reductions in medical errors, time savings) BSC: Internal Process	Guideline: Establish coordination mechanisms that actively include stakeholders Indicator: Stakeholder engagement metrics (e.g., participation rates, satisfaction) BSC: Patient
Institutional Positioning	Guideline: Incorporate strategic achievements into external institutional messaging Indicator: Brand perception metrics (e.g., recognition of strategic excellence) BSC: Financial	Guideline: Highlight collaborative capabilities in communications with external partners Indicator: Partnership development metrics (e.g., new relationships, referral rates) BSC: Financial	Guideline: Cultivate stakeholder trust through consistent transparent communication Indicator: Trust and reputation metrics (e.g., stakeholder satisfaction, loyalty) BSC: Financial/Patient

### 6.1 Theoretical foundation and purpose

The proposed matrix synthesizes the four communicative flows from CCO theory with our healthcare-adapted corporate communication dimensions to create a comprehensive assessment tool. This integration bridges micro-level communicative interactions that constitute organizational reality with macro-level strategic communication management processes essential in healthcare contexts.

This evaluation framework serves multiple purposes for healthcare organizations:

1. Identify gaps in their current BSC communication practices.
2. Develop targeted strategies to enhance communication effectiveness.
3. Align communication practices with strategic objectives.
4. Track progress in communication improvement over time.

### 6.2 Healthcare BSC communication matrix

As shown in Table 2, the matrix is structured as a 4 × 3 grid that maps the four communicative flows (membership negotiation, organizational self-structuring, activity coordination, and institutional positioning) against the three healthcare communication dimensions (clinical strategic communication, interprofessional coordination communication, and healthcare stakeholder communication).

Each cell in the matrix contains:

- A guideline that describes recommended communication practices.
- An indicator that suggests metrics for measuring communication effectiveness.
- A BSC that links the communication practice to the organization’s BSC.

The matrix (Table 2) is designed to be used with specific strategic objectives, allowing healthcare organizations to tailor their communication assessment to their unique priorities and goals.

### 6.3 Application process

To effectively implement this evaluation framework, we recommend healthcare organizations follow these steps:

1. Strategic objective definition: Begin by clearly articulating a specific strategic objective in the designated field at the top of the matrix. This strategic objective should derive from the healthcare values foundation layer of our multilayered model (Figure 1), ensuring alignment with its six interconnected components (i.e., mission, vision, organizational values, clinical values, social responsibility, stakeholder expectations, and global standards). For example, an objective might be “Implement an electronic medical record system that enhances interprofessional collaboration while maintaining our commitment to compassionate, patient-centered care.”
2. Assessment of current state: For each cell in the matrix, evaluate current communication practices against the provided guidelines. This assessment can be conducted through structured surveys, focus groups, document analysis, or observational studies.
3. Identification of communication gaps: Compare current practices with the recommended guidelines to identify specific areas where communication could be strengthened.
4. Development of communication strategies: Based on identified gaps, develop targeted communication strategies for high-priority areas. These strategies should specify:
  - Communication channels and formats
  - Key messages and narratives
  - Responsibility assignments
  - Implementation timelines
5. Measurement and evaluation: Implement the suggested indicators to establish baseline measurements and track improvements over time. These metrics should be incorporated into the organization’s BSC reporting system, creating a feedback loop that connects back to the healthcare communication processes and constitutive processes layers of our framework (Figure 1).

### 6.4 Application of the evaluation framework: electronic medical record implementation

To illustrate how this matrix might be applied, we revisit the EMR implementation at Bethlehem Garden Hospital, discussed in Section 5. For this demonstration, we apply our Healthcare BSC Communication Matrix to analyze how the hospital’s communication practices aligned with the strategic objective of implementing an EMR system to enhance interprofessional collaboration.

Table 3 shows a hypothetical assessment based on the documented case. Note that this assessment was constructed by applying our framework to the already documented evidence rather than through direct observation or additional data collection.

**Table 3:** Illustrative example: EMR implementation assessment [source: compiled by the authors].

Communication focus	Current practice	Assessment	Improvement opportunity
Clinical Strategic Communication × Membership Negotiation	Regular presentations by Director on strategic context of EMR implementation	Moderate	Develop more personalized narratives connecting EMR to specific professional roles and values
Interprofessional Coordination × Organizational Self-structuring	Established project meeting structure with representatives from all departments	Strong	Further formalize cross-departmental information sharing protocols beyond project team
Healthcare Stakeholder Communication × Activity Coordination	Limited patient involvement in EMR implementation process	Weak	Develop mechanisms for incorporating patient feedback in EMR workflow design

This assessment reveals several key insights:

1. Strengths identified: The hospital demonstrated particularly strong interprofessional coordination communication, as evidenced by their cross-departmental project team structure and collaborative workflow development.
2. Areas for improvement: The analysis identified gaps in healthcare stakeholder communication, particularly in patient involvement during the implementation process.
3. Practical implications: Based on this assessment, the hospital might prioritize:
  - Developing structured mechanisms for incorporating patient perspectives in technology implementations
  - Creating formal channels for collecting and integrating patient feedback
  - Establishing regular stakeholder engagement activities to ensure all voices are heard

This example demonstrates how healthcare organizations can systematically evaluate their communication practices using the proposed matrix to identify specific areas for improvement and develop targeted intervention strategies.

## 7 Conclusions and future directions

This paper has developed a comprehensive theoretical framework for analyzing BSC as a communication tool in healthcare settings, building on our previous work (15) that established the BSC’s effectiveness as a corporate communication tool in a Japanese hospital context. By integrating CCO theory’s four flows model with a healthcare-adapted corporate communication framework, we have created a multilayered analytical approach that addresses the specific communication challenges faced by healthcare organizations.

While our previous research (15) adapted Van Riel and Fombrun’s (16) traditional corporate communication framework to analyze BSC at Bethlehem Garden Hospital, this modification remained within the bounds of generic corporate communication rather than developing a truly healthcare-specific model. This paper makes a substantial theoretical advancement by creating a comprehensive framework that integrates healthcare-specific communication dimensions with constitutive processes, capturing the distinctive communication dynamics inherent in clinical environments. By moving beyond conventional corporate communication categories to develop healthcare-specific communication dimensions, we provide a more nuanced understanding of the unique challenges and opportunities faced by healthcare organizations in strategy implementation.

The integrated framework proposes several key theoretical advances beyond our previous research. First, it shows how healthcare values serve as the foundation for BSC implementation, informing both the design of performance measures and the communication processes that support them. When key values, such as organizational values like “compassionate care” or social responsibility values like “community service,” are translated into specific BSC indicators, they become operational priorities rather than merely aspirational statements.

Second, the framework reveals the dynamic bidirectional relationships between framework layers that create continuous feedback loops, enhancing BSC effectiveness. The analysis of these interactions demonstrates how BSC indicators provide structured content for organizational communication, while communication processes determine how this content is effectively disseminated and internalized across diverse professional groups.

Third, the multilayered nature of the framework illuminates how BSC initiatives can operate simultaneously across multiple communication dimensions and constitutive flows. This integrated perspective reveals how BSC transforms abstract healthcare values into measurable objectives, coordinates improvement efforts across disciplinary boundaries, and communicates progress to diverse stakeholders. The practical application of this theoretical understanding is demonstrated in our proposed Healthcare BSC Communication Matrix (Table 2), which provides healthcare leaders with specific guidelines and metrics for enhancing their organizational communication through BSC implementation.

For healthcare leaders, this enhanced framework offers practical guidance for implementing the BSC more effectively. By recognizing the complex interactions between healthcare values, performance measures, communication processes, and constitutive mechanisms, organizations can design BSC implementations that enhance both performance management and organizational communication quality. This integrated perspective enables healthcare leaders to move beyond viewing BSC as merely a measurement system to recognizing its power as a communication tool that can transform organizational dynamics in ways that ultimately improve patient care.

### 7.1 Limitations

While our framework and proposed evaluation matrix provide valuable insights for both theory and practice, we acknowledge several limitations:

1. Contextual variation: The applicability of our framework may vary based on organizational size, structure, and cultural context. Healthcare organizations operate in diverse environments with different regulatory requirements, funding models, and cultural expectations, which may influence how BSC functions as a communication tool.
2. Measurement challenges: Some of the proposed indicators in our evaluation matrix may be difficult to operationalize consistently across organizations. The subjective nature of communication quality assessment presents inherent challenges in developing standardized metrics.
3. Empirical validation: As a theoretically derived framework, both our multilayered model and evaluation matrix require empirical validation across diverse healthcare settings to confirm their effectiveness and refine their components. Our illustrative case provides preliminary support for the framework’s applicability, but more comprehensive testing is needed.
4. Scope limitations: This study primarily focuses on a long-term care hospital and does not address in detail the specific challenges and opportunities in other healthcare settings such as acute care hospitals, primary care facilities, or specialty hospitals.
5. Geographic and cultural context: Our framework draws primarily on experiences from East Asian healthcare environments, particularly our case study of a Japanese hospital. Caution should be exercised when generalizing to environments with different healthcare systems, cultural backgrounds, or organizational structures. Cultural factors such as power distance, communication styles, and decision-making processes may influence how BSC functions as a communication tool. For example, Abu Jaber and Nashwan’s (55) successful BSC implementation in Middle Eastern healthcare settings, with different cultural and organizational contexts than those in Japan, demonstrates the need for cultural adaptation when applying BSC communication frameworks across diverse healthcare environments.

### 7.2 Future research directions

These limitations suggest several promising directions for future research:

1. Multi-site comparative studies: Future studies should test the framework’s applicability across diverse healthcare settings, including different types of healthcare organizations (such as primary care, specialized hospitals, and long-term care facilities) and various national healthcare systems. Such comparative research could identify contextual factors that influence the effectiveness of BSC as a communication tool.
2. Measurement tool development: Researchers should develop and validate standardized assessment instruments for measuring the communication dimensions identified in our framework. These tools would enable more rigorous empirical testing of the relationships between BSC implementation, communication quality, and organizational outcomes.
3. Longitudinal studies: Exploring the long-term impacts of BSC-facilitated communication on organizational performance, staff engagement, and patient outcomes would provide valuable insights into the sustainability and evolving nature of communication practices in healthcare organizations.
4. Integration with clinical outcomes: Future research should examine the relationship between improvements in BSC communication practices and specific clinical outcomes, establishing more direct links between communication effectiveness and patient care quality.
5. Cultural adaptations: Exploring how cultural factors influence the implementation and effectiveness of BSC as a communication tool in different national contexts would enhance the framework’s global applicability.
6. Training programs: Future studies could investigate how to design training programs tailored specifically to this integrated framework. This is a key research direction, as the required training would likely differ from that discussed in our previous research (15). Our current framework’s integration of micro-level CCO theory with the macro-level corporate communication perspective suggests that effective training must address both strategic communication management and constitutive processes that foster shared understanding across professional boundaries.

Despite these limitations, this paper makes an essential contribution to both theory and practice by reconceptualizing the BSC as a constitutive communication mechanism rather than merely a measurement tool in healthcare settings. The multilayered model and practical evaluation matrix provide healthcare leaders with new perspectives and tools for enhancing organizational communication through more effective BSC implementation. By bridging theory and practice, this research aims to ultimately improve healthcare organization performance and, consequently, the quality of patient care.

## Data Availability

The original contributions presented in the study are included in the article/supplementary material, further inquiries can be directed to the corresponding author.
